# Comparing effects of soybean oil- and palm olein-based mayonnaise consumption on the plasma lipid and lipoprotein profiles in human subjects: a double-blind randomized controlled trial with cross-over design

**DOI:** 10.1186/s12944-016-0301-9

**Published:** 2016-08-17

**Authors:** Tilakavati Karupaiah, Khun-Aik Chuah, Karuthan Chinna, Ryosuke Matsuoka, Yasunobu Masuda, Kalyana Sundram, Michihiro Sugano

**Affiliations:** 1 Dietetics Program, School of Healthcare Sciences, Faculty of Health Sciences, National University of Malaysia, Kuala Lumpur, Malaysia; 2 Nutrition Program, School of Healthcare Sciences, Faculty of Health Sciences, National University of Malaysia, Kuala Lumpur, Malaysia; 3 Julius Center, Department of Social and Preventive Medicine, Faculty of Medicine, University of Malaya, Kuala Lumpur, Malaysia; 4 R&D Division, Kewpie Corporation, Sengawa Kewport, 2-5-7, Sengawa-cho, Chofu-shi, Tokyo, Japan; 5 Malaysian Palm Oil Council, Kelana Jaya, Selangor Malaysia; 6 Kyushu University, and Prefectual University of Kumamoto, Kyushu, Japan

**Keywords:** Mayonnaise, Fatty acids, Cardiometabolic risk, Lipids, Lipoprotein particles

## Abstract

**Background:**

Mayonnaise is used widely in contemporary human diet with widespread use as a salad dressing or spread on breads. Vegetable oils used in its formulation may be a rich source of ω-6 PUFAs and the higher-PUFA content of mayonnaise may be beneficial in mediating a hypocholesterolemic effect. This study, therefore, evaluated the functionality of mayonnaise on cardiometabolic risk within a regular human consumption scenario.

**Methods:**

Subjects underwent a randomized double-blind crossover trial, consuming diets supplemented with 20 g/day of either soybean oil-based mayonnaise (SB-mayo) or palm olein-based mayonnaise (PO-mayo) for 4 weeks each with a 2-week wash-out period. The magnitude of changes for metabolic outcomes between dietary treatments was compared with PO-mayo serving as the control. The data was analyzed by ANCOVA using the GLM model. Analysis was adjusted for weight changes.

**Results:**

Treatments resulted in significant reductions in TC (diff = −0.25 mmol/L; *P =* 0.001), LDL-C (diff = −0.17 mmol/L; *P* = 0.016) and HDL-C (diff = −0.12 mmol/L; *P* < 0.001) in SB-mayo compared to PO-mayo without affecting LDL-C:HDL-C ratio (*P* > 0.05). Lipoprotein particle change was significant with large LDL particles increasing after PO-mayo (diff = +63.2 nmol/L; *P* = 0.007) compared to SB-mayo but small LDL particles remained unaffected. Plasma glucose, apolipoproteins and oxidative stress markers remained unchanged.

**Conclusions:**

Daily use with 20 g of linoleic acid-rich SB-mayo elicited reductions in TC and LDL-C concentrations without significantly changing LDL-C:HDL-C ratio or small LDL particle distributions compared to the PO-mayo diet.

**Trial registration:**

This clinical trial was retrospectively registered with the National Medical Research Register, National Institute of Health, Ministry of Health Malaysia, (NMRR-15-40-24035; registered on 29/01/2015; https://www.nmrr.gov.my/fwbPage.jsp?fwbPageId=ResearchISRForm&fwbAction=Update&fwbStep=10&pk.researchID=24035&fwbVMenu=3&fwbResearchAction=Update). Ethical approval was obtained from the National University of Malaysia’s Medical Ethics Committee (UKM 1.5.3.5/244/SPP/NN-054-2011, approved on 25/05/2011).

## Background

Therapeutic lifestyle changes that address dietary correction are core principles to prevent chronic disease burden. The United States Dietary Guidelines (USDG) Technical Report in 2015 [1] dropped dietary cholesterol from the list of ‘nutrients of concern’ in relation to the chronic disease burden. This update is in concordance with the lack of evidence from clinical studies showing a benefit in dietary cholesterol reduction [2]. Instead, the USDG Technical Report has emphasized substituting saturated fats with polyunsaturated fatty acid (PUFA) alternatives as part of healthy dietary recommendations [1]. Based on controlled feeding trials, the strength of evidence is rated as strong for every 1 % of energy from saturated fatty acid (SFA) that is replaced with PUFA which produces a 1.8 mg/dL decrease in low density lipoprotein-cholesterol (LDL-C) [3]. The consumption of PUFA-enriched foods, mainly from the ω-6 family, is therefore necessary in order to make this recommendation actionable. The relationship between SFA and PUFA intakes in general populations, shows that lower intakes of SFA are not accompanied by higher intakes of PUFA, as is recommended for preventing coronary heart disease [4]. In this regard, the nutraceutical actions of functional food ingredients are been explored as either adjuvant or alternate therapy to pharmacological interventions [5].

PUFA availability in the human diet is further differentiated into ω-3 and ω-6 fatty acid classes. The ω-3 PUFAs are involved in prostaglandin E3, prostaglandin I3 and thromboxane A3 production while ω-6 PUFAs are involved in the production of prostaglandin E2, prostaglandin I2 and thromboxane A2; and these two fatty acid classes are known to exhibit antagonistic effects. Looking at effects on serum lipids, it has been reported that ω-3 PUFAs lower triglycerides thereby benefiting dyslipidemia management whereas ω-6 PUFAs, specifically linoleic acid, cause decreased LDL-C concentrations [5]. Global data on country-specific ω-6 consumption ranges between 1.2 and 12.5 % of energy, with a global mean of 5.9 % energy [6]. Therefore, in practice, there is dietary insufficiency of ω-6 PUFA in the human diet in most countries, and the recommendation to increase PUFA for cardiovascular disease (CVD) prevention requires some degree of dietary supplementation. In contrast, although ω-3 PUFAs have a secondary prevention role in CVD mortality, it is ω-6 PUFA that is perceived to have an etiological effect as a replacement for SFA in reducing LDL-C [6]. A long-term prospective cohort running for 32 years showed ω-6 PUFA intake, especially linoleic acid, was inversely associated with mortality from major CVD causes [7].

Mayonnaise is a mainstay in the contemporary human diet with widespread use as a salad dressing in the home or restaurant. Traditional mayonnaise is a source of ω-6 PUFAs depending on the source of compositional oils which may be from soybean, canola, corn or sunflower sources [8]. The functional role of fat in mayonnaise formulation is in maintaining food quality related to texture, flavor, and stability of food emulsion products [9]. The PUFA content of mayonnaise ranges between 28.0 and 47.9 g/100 g product [10]. Most regular mayonnaise products produced commercially are formulated with soybean oil (SB). SB contains a significant proportion of linoleic acid (LA, 18:2 > 50 %) and less palmitic acid (16:0 < 10 %) and being PUFA-rich would be expected to contribute to a hypocholesterolemic effect [11]. However if this is true, no literature has reported on the cholesterolemic lowering effect of mayonnaise if consumed consistently over a period of time. On the other hand, Amini et al. [12], who conducted a cross-sectional examination of adult Iranian diets (*n* = 425), suggested mayonnaise as part of an undesirable Western-pattern diet matrix was associated with risk of metabolic syndrome (MetS). This view differs from a Japanese study which in examining associations between major dietary patterns and MetS prevalence, reported mayonnaise was not associated with prevalence of MetS, but was positively associated with high blood glucose [13].

Although, coronary risk is assessed in terms of atherosclerosis development [3], the wider scope today includes MetS, with inflammation and oxidative stress implicated in its early pathology [14–16]. Our key research question therefore was- given the fatty acid compositional profile of mayonnaise if consumed on a daily basis- what would be its direct impact on lipid and lipoprotein profile? On a wider scope, what would be the impact on other metabolic parameters related to oxidative stress and inflammation? An additional interest in this study was to also address an issue whether subclass differences in LDL and high density lipoprotein (HDL) particle profiles were responsive to the manipulated linoleic acid content of the total diet.

## Methods

### Subject demographics

The study was conducted at a teachers’ training institute and subjects were recruited via the institutional noticeboard. Initially 36 healthy Malaysian subjects without a history of atherosclerotic disease, diabetes or hypertension were recruited but 2 subjects withdrew voluntarily in the first week of the trial as they were unwilling to conform to the study protocol. Finally, 16 men and 18 women participated with mean prescreening plasma total cholesterol (TC) levels of 5.01 ± 0.54 mmol/L but classified into 2 groups – either normocholesterolemic values (*n* = 21, <5.2 mmol/L) or with mild/borderline hypercholesterolemia (*n* = 13, 5.2-6.0 mmol/L). Overall mean (±SD) characteristics for subjects who qualified, signed informed consent and completed the study were: age = 23.36 ± 6.98 year, body weight = 64.49 ± 14.41 kg and body mass index (BMI) = 25.08 ± 4.71 with plasma values for TC = 5.01 ± 0.54 mmol/L, HDL-C =1.39 ± 0.30 mmol/L, LDL-C = 3.18 ± 0.48 mmol/L, triacylglycerol (TAG) = 0.99 ± 0.46 mmol/L and fasting glucose = 4.70 ± 0.44 mmol/L (Table 1).

**Table 1:** Subject characteristics

Characteristics	Overall (*n* = 34)
Gender (M/F)	16/18
Age (yr)	23.4 ± 7.0
Body weight (kg)	64.5 ± 14.4
BMI (kg/m^2^)	25.1 ± 4.7
TC (mmol/L)	5.01 ± 0.54
HDL-C(mmol/L)	1.39 ± 0.30
LDL-C(mmol/L)	3.18 ± 0.48
TAG (mmol/L)	0.99 ± 0.46
Glucose (mmol/L)	4.70 ± 0.44

All blood parameters are based on plasma analysis; Values represent mean ± SD of 34 subjects

*Abbreviations*: *BMI* body mass index, *TC* total cholesterol, *HDL-C* high density lipoprotein-cholesterol, *LDL-C* low density lipoprotein-cholesterol, *TAG* triacylglycerol

### Study design

**Fig. 1:**
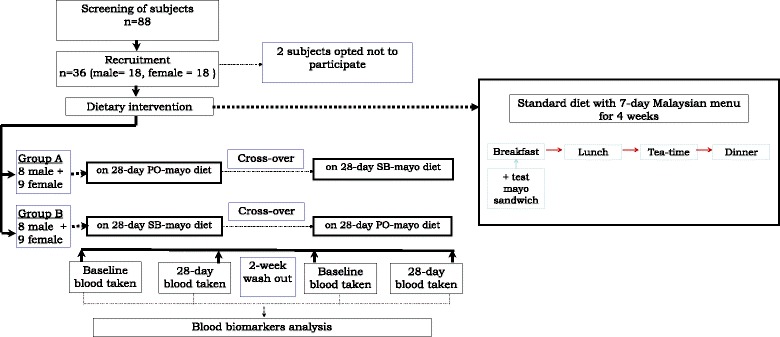
The flow chart for the study design

A 2-period cross-over design with a 2-week wash-out between rotations of 4 weeks each was adopted for the study. Subjects were randomized between 2 groups of 17 each with both groups on parity for gender, age and plasma TC levels. For each 4-week rotation, one group received as test a soybean-based mayonnaise product (SB-mayo) with the background diet whilst the other group received a palm olein-based mayonnaise product (PO-mayo) serving as a control with the similar background diet. The purpose of using a PO-based mayo in the control diet was to optimize it as a habitual diet reflective of a higher SFA content. The cross-over of diet treatments took place after completion of the 2-week wash-out period following the first 4-week feeding period. In the 2-week wash-out period subjects were allowed to return to free living conditions and consume their habitual diets *ad libitum*. Researchers and subjects were blinded to test mayonnaise products which were labeled as Mayo A or Mayo B. Only the study dietitian serving as the controller knew the mayonnaise codes and was responsible for assigning the sandwiches made with test mayonnaise to the respective groups. Figure 1 indicates the flow chart for this study.

This study was conducted in accordance with the principles of the Declaration of Helsinki with ethical approval provided by the National University of Malaysia’s Medical Ethics Committee (UKM 1.5.3.5/244/SPP/NN-054-2011 approved on 25/05/2011).

### Test mayonnaise products

The amount of mayonnaise planned for daily consumption during this trial was based on a test fat load required to elicit lipid and lipoprotein changes in an overall daily diet deriving ~30 %-en from total dietary fats as reported in our previous studies [17–19]. The calculated fat was provided by an equivalent of ~20 g mayonnaise per subject for each trial feeding day and when incorporated into sandwiches resulted in an estimated 15 g of respective test fat load delivery. These sandwiches were made by the study dietitian serving as the controller, who was responsible for assigning the sandwiches as per test mayonnaise code to the subjects. The completely formulated test product (SB-mayo) was supplied in pre-packed sachets whereas the control (PO-mayo) was provided only as egg slurry similar in composition to the SB-mayo (R&D Division Kewpie Corporation, Japan, and Kewpie Malaysia Sdn. Bhd, Malaysia). The control egg slurry was emulsified daily by the study dietitian with an amount of palm olein equivalent to the soybean oil content in the SB-mayo. This daily emulsification process was necessary as otherwise this mayonnaise product could not hold stability. Table 2 indicates the percent fatty acid composition (FAC) of the 2 mayonnaise products used in the study. Only statistically significant differences (*P* < 0.05) between the 2 products were related to palmitic, oleic, linoleic and linolenic acid content.

**Table 2:** Percent fatty acid composition (%) of mayonnaise products, duplicated diets and plasma triacylglycerol

Fatty acids (%)	Mayonnaise products		Duplicated total diets ^b^		Plasma triacylglycerol		
	SB-mayo	PO-mayo	SB-mayo	PO-mayo	Baseline	SB-mayo day 28	PO-mayo day 28
C12:0	0.03	0.29	0.37 ± 0.29	0.51 ± 0.41	0.43 ± 0.06	0.49 ± 0.35	0.36 ± 0.64
C14:0	0.07	0.90	0.79 ± 0.12	0.95 ± 0.35	1.57 ± 0.70	1.14 ± 0.47	0.99 ± 0.41
C16:0	10.4	37.7	28.0 ± 2.9*	34.6 ± 1.7*	32.1 ± 2.3	29.6 ± 1.7*	31.2 ± 1.7*
C16:1ω7	0.15	0.16	0.55 ± 0.12	0.62 ± 0.17	4.09 ± 0.81	4.26 ± 0.87	4.21 ± 1.11
C18:0	3.51	2.78	4.40 ± 0.93	4.23 ± 0.52	4.09 ± 1.17	3.16 ± 0.64	3.08 ± 0.52
C18:1	24.3	45.1	37.5 ± 1.2*	41.8 ± 1.7*	40.9 ± 2.3	40.9 ± 2.3*	43.7 ± 1.7*
C18:2ω6	53.1	12.3	23.4 ± 3.5*	13.0 ± 1.7*	12.0 ± 1.7	15.3 ± 1.2*	12.7 ± 1.7*
C18:3ω6	nd	nd	0.18 ± 0.12*	0.04 ± 0.35*	0.24 ± 0.12	0.20 ± 0.12	0.15 ± 0.06
C18:3ω3	5.56	0.29	1.05 ± 0.41	0.77 ± 0.87	0.30 ± 0.12	0.35 ± 0.12	0.26 ± 0.17
C20:4ω6	nd	nd	0.10 ± 0.58	0.15 ± 0.93	0.57 ± 1.98	0.62 ± 0.23	0.59 ± 0.23
Total SFA	14.0	41.7	35.1 ± 2.8*	41.9 ± 1.3*	37.9 ± 3.3	34.3 ± 2.4*	35.5 ± 2.2*
Total MUFA	24.5	45.3	38.5 ± 1.3*	42.8 ± 1.8*	45.0 ± 2.5	45.1 ± 2.6*	47.9 ± 2.0*
Total PUFA	58.7	12.6	25.7 ± 3.6*	14.4 ± 0.9*	12.9 ± 2.3	16.3 ± 1.4*	13.4 ± 1.9*
Total ω-6	53.1	12.3	24.0 ± 3.7*	13.2 ± 1.0*	12.7 ± 2.2	15.5 ± 3.1*	13.1 ± 1.8*
Total ω-3	5.56	0.29	1.05 ± 0.41	0.77 ± 0.87	0.28 ± 0.14	0.35 ± 0.13	0.26 ± 0.15
ω-6:ω-3 ratio	9.55	42.4	26.7 ± 11.9	24.2 ± 9.6	48.0 ± 14.2	55.7 ± 31.5	71.4 ± 71.4
id	97.12	99.52	96.34	96.67	96.29	96.02	97.24
^a^ unid	2.88	0.48	3.66	3.33	3.71	3.98	2.76

*Abbreviations*: *C* carbon chain length, *MUFA* mono-unsaturated fatty acids, *SB-mayo* soybean oil-based mayo, *PO-mayo* palm olein-based mayo, *PUFA* poly-unsaturated fatty acids, *nd* not detected, *id* identified, *SFA* saturated fatty acids, *unid* unidentified, *ω-6* omega 6, *ω-3* omega 3

^a^percent unidentified fatty acids constitute <4 % of total fatty acid composition

^b^Represents total daily diet planned as per a research menu and FAC analyses is performed from duplicate diets collected from subjects. Values represent mean ± SD

*significance shown with *P* < 0.05

### Test diets

As study subjects were residing on campus in the study site, all meals inclusive of breakfast, lunch, tea and dinner for each fat rotation were provided by the institutional caterer as instructed by the research dietitian. Subjects were expected to consume these meals at a designated residential cafeteria supervised by the dietitian. A standardized 7-day cycle menu planned with a fixed daily total fat and cholesterol content was used for the two 4-week test rotations. All meals were cooked with palm olein to fix the background fat for meal preparation and were standardized to recipes, portion sizes and procedures for incorporating the cooking oil. The amount of cooking oil used was enough to maintain a reduced fat background (~25 %-en) allowing subjects in the 2 test rotations ~45 g per day of palm olein whilst ~15 g of fat was derived from the test mayonnaise during the feeding trial. Other sources of SFAs such as lauric and myristic acids were limited in the menu by excluding coconut-based products and full-fat dairy products. Skim milk and a limit of 2 eggs per week were allowed. The daily diets provided a total of 62–64 g of fat; the SB-mayo diet carrying 12 g of linoleic acid (~1/3^rd^ of total fat) from the mayonnaise yielded final concentrations of 23.4 % 18:2ω6 and 1.05 % 18:3ω3 vs 13.0 % 18:2ω6 and 0.77 % 18:3ω3 in the PO-mayo based diet. Thus for the total diet comparisons, the total ω6 was significantly higher in SB-mayo compared to PO-mayo treatment. Subjects were instructed to eat according to their usual capacity for food intake as determined by energy intake assessment from 24-h diet records at baseline. This pre-study energy intake assessment provided a guideline to the dietitian for ensuring monitoring subjects’ weight fluctuations did not exceed 1 kg within each 4-week test rotation.

### Compliance measures

For each rotation these included [i] meal time attendance records [ii] weekly recording of body weights of subjects [iii] collecting 3 × 24-h diet records per rotation from every subject and [iv] preparing duplicated diets of all foods consumed over a 24-h period including snacks and fluids except water from subjects for FAC analysis. The 24-h diet records were analyzed with NutriPro software [version 3.2.0] which includes databases for Malaysian foods [20].

### Blood collection and analyses

All subjects arrived at the laboratory after an overnight fasted state (~10 h) on testing days at baseline, day 27 and day 28 of each test rotation. Blood was collected into Vacutainer® tubes (Becton Dickinson Vacutainer, NJ, USA) containing EDTA (0.117 ml of 15 % EDTA) and immediately centrifuged at 3000 g for 10 min at 4 °C (Sigma 3 K12 B.Braun, Germany) to separate plasma from red blood cells. Plasma aliquots were snap-frozen in liquid nitrogen and stored at −80 °C for analyses after study completion. To reduce intra-variations in the analysis, plasma samples were analyzed in a single batch.

#### Analyses of lipids and lipoproteins

Analyses were carried out using automated clinical chemistry procedures. Enzymatic kits were used to measure plasma TC, TAG and HDL-C (Roche Diagnostics Corporation, Indianapolis, IN, USA). For the determination of cholesterol and TAG concentrations the assay procedures were based on Allain et al. (1974) and Nägele et al. (1984), respectively [21, 22]. The Cobas Integra HDL-Cl plus 2^nd^ generation in vitro diagnostic kit, for the direct determination of HDL-C, combines polyethylene glycol-modified enzymes with sulphated α-cyclodextrins [23]. These assays were performed using a Cobas 6000 Chemistry Autoanalyzer System (Roche Analytic Instruments Inc, Nutley, NJ, USA). Reagents, calibrators and controls were also supplied by the manufacturer (Roche Diagnostics Corporation, Indianapolis, IN, USA). LDL-C was calculated using the Friedewald equation [24]. Very low density lipoprotein-cholesterol (VLDL-C) was calculated by multiplying TAG values by a factor of 0.46.

#### Determination of glucose

Plasma glucose was assessed by the hexokinase –glucose-6-phosphate dehydrogenase 2-step enzymatic procedure [25]. An in vitro diagnostic reagent kit for this enzymatic procedure, Glucose HK Liquid (GLUCL 0–992) (Roche Diagnostics Corporation, Indianapolis, IN, USA) was used for automated analysis (Roche Analytic Instruments Inc, Nutley, NJ, USA).

#### Determination of non-esterified fatty acids (NEFA)

Plasma NEFA was assessed by the acyl-CoA synthase-acyl CoA oxidase-peroxidase (ACS-ACOD) 3-step enzymatic procedure [26] using a commercial kit, Lab Assay™ NEFA (Wako pure chemical industries Ltd., Osaka, Japan) with absorbance detected at 550 nm by a multi-detection microplate reader (Powerscan® HT, DS Pharma Biomedical Co. Ltd., Osaka, Japan).

#### Determination of apolipoproteins (apo) A1 and B

Plasma apolipoproteins were assessed by the sandwich ELISA procedure using anti-human apoA1 or apoB monoclonal antibody, and horseradish peroxidase-labeled anti-human apoA1 or apoB monoclonal antibody, respectively [27]. A commercial kit, ELISA^pro^ kit for Human ApoA1, or ApoB [Mabtech Inc., OH, USA] was used with absorbance detected at 450 nm by a multi-detection microplate reader [Powerscan® HT, DS Pharma Biomedical Co. Ltd., Osaka, Japan].

#### Determination of thiobarbituric acid reactive substances (T-BARs)

T-BARs was assessed by the malondialdehyde-TBA adduct formed by the reaction of malondialdehyde and T-BARs [28] under high temperature (90–100 °C) using a commercial kit, T-BARs assay kit (Cayman Chemical Company, MI, USA). Acidic conditions were detected by measuring absorbance at 550 nm with a multi-detection Microplate reader (Powerscan® HT, DS Pharma Biomedical Co. Ltd., Osaka, Japan).

#### Determination of high sensitivity C-reactive protein (hsCRP)

Plasma hsCRP was assessed by a sandwich ELISA protocol with a commercial kit (Helica Biosystems Inc., CA, USA) which uses anti-human CRP monoclonal antibody, and horseradish peroxidase-labeled anti-human CRP monoclonal antibody [29]. Absorbance at 450 nm was detected by a multi-detection microplate reader (Powerscan® HT, DS Pharma Biomedical Co. Ltd., Osaka, Japan).

#### NMR lipoprotein particle measures

Particle count and particle size profile determinations for LDL and HDL were carried out by automated nuclear magnetic resonance (NMR) spectroscopic assay by an independent laboratory (LipoScience Inc., Raleigh, North Carolina, USA). EDTA plasma samples held at −80 °C at study conclusion were shipped out in dry ice to the certified laboratory (CLIA ID No. 34D0952253) as per service provider instructions. The NMR technology streamlines the time-consuming ultracentrifugation process into a 2-step analytical process using automated NMR signal acquisition followed by computational analysis [30].

#### Analyses of fatty acid composition

Test mayonnaise, duplicated diets and total plasma were subjected to FAC analysis using methodology as described previously [17–19]. FAC analysis was carried out by means of gas chromatography (Shimadzu Scientific Instruments, Japan). The fatty acid profile of analyzed meals served to check whether research diets achieved fatty acid targets. FAC determination of plasma TAG served to check subjects’ compliance to targeted mayonnaise consumption in each test fat rotation as per research protocol.

### Statistical analysis

A cross-over design was used which enabled subjects to serve as their own control. For each subject, the mean value for day 27 and day 28 in each treatment rotation was taken as the end point. In descriptive statistics, data were expressed as mean ± SD and the level of significance was set as *P* < 0.05. We deduced that the 2-week washout period between treatments was sufficient by comparing values for the 2 baselines by paired sample *t*-test analysis and found a lack of significance (*P* > 0.05). Within and between treatment comparisons of targeted fatty acids in plasma triglycerides expected to be reflective of mayonnaise treatment were analyzed by ANCOVA analysis using GLM model. Weight change between treatment periods was analyzed for statistical significance on metabolic outcomes. However, all analysis to test difference in means for metabolic outcomes were carried out controlled for weight changes by ANCOVA procedure using GLM model. The magnitude of changes for metabolic outcomes between dietary treatments was compared with PO-mayo serving as the control for comparison and data was analyzed by ANCOVA using the GLM model. The Statistical Package for Social Sciences, SPSS® for Windows™ application (Version 18.0, SPSS Inc., Chicago, USA) was used for all analyses.

## Results

### Measures of compliancy

#### Weight monitoring

Weight reductions occurred for both rotations (SB-mayo = −0.6 ± 1.1 kg, *P* = 0.003 and PO-mayo = − 0.9 kg ± 1.1 kg, *P* < 0.001) compared to baseline. However, mean weight loss at the end of the study periods was less than 1 kg and not significantly different between groups (*P* > 0.05). This weight change did not influence metabolic outcomes or lipoprotein particle size distributions (*P* > 0.05).

#### FAC analysis of diets and plasma TAG

Targeted palmitic, oleic, linoleic and linolenic acids of duplicated diets collected from subjects during each test rotation were significantly different (*P* < 0.001) between SB-mayo and PO-mayo diets (Table 2). Similarly, consumption of these diets reflected in within- and between-diet effects specific to palmitic, oleic and linoleic acids that were significantly different (*P* < 0.05) as indicated by FAC analysis of plasma TAG (Table 2). No difference in arachidonic acid (%) in plasma TAG was apparent between dietary treatments (*P* > 0.05).

#### Nutrient intake

Subjects’ consumption of calories, protein, carbohydrate, fat, cholesterol and fiber were similar (*P* > 0.05) in both arms of the study but only SFA, monounsaturated fatty acid (MUFA) and PUFA content were significantly different (*P* < 0.001) between test rotations (Table 3).

**Table 3:** Nutrient composition of consumed diets per day

Nutrients	Dietary recalls ^a^	
	SB-mayo	PO-mayo
Energy (kcal)	1938 ± 279	1994 ± 356
Protein (g)	57.6 ± 11.7	58.9 ± 14.0
CHO (g)	284 ± 52	293 ± 64
Fat (g)	62.2 ± 8.2	64.1 ± 9.9
Cholesterol (mg)	147 ± 41	151 ± 41
SFA (%)	7.8 ± 1.4*	10.9 ± 1.8*
MUFA (%)	9.4 ± 1.2*	11.6 ± 1.8*
PUFA (%)	12.0 ± 2.2*	6.8 ± 1.8*
Fiber (g)	6.27 ± 1.63	6.58 ± 2.86

*Abbreviations SB-mayo* soybean oil-based mayo, *PO-mayo* palm olein-based mayo, *CHO* carbohydrate, *SFA* saturated fatty acids, *MUFA* monounsaturated fatty acids, *PUFA* polyunsaturated fatty acids

*significance shown with *P* < 0.05

^a^Diet recall nutrient values (mean ± SD of 34 subjects) were based on 3 × 24-h diet recalls collected for each subject for each test rotation and calculated from a reference nutrient database (First Databank, 2005)

### Metabolic outcomes

Changes in lipid and lipoprotein parameters as a result of manipulating the mayonnaise content of the diet occurred as indicated in Table 4. After adjustment for baseline, effects attributable to dietary treatment alone were:

i. SB-mayo treatment significantly reduced TC compared to the PO-mayo diet (diff = −0.25 ± 0.40 mmol/L; *P =* 0.001).
ii. LDL-C reduction was significantly greater after the SB-mayo treatment compared to the PO-mayo treatment (diff = −0.17 ± 0.40 mmol/L; *P* = 0.016).
iii. Reduction in HDL-C concentration was associated with the SB-mayo dietary treatment which was significantly lower from the PO-mayo treatment (diff = −0.12 ± 0.15 mmol/L; *P* < 0.001).
iv. Changes in LDL-C:HDL-C ratio between diets were not significant (*P* > 0.05).
v. Plasma TAG, VLDL-C and glucose changes between dietary treatments were not significant (*P* > 0.05).
vi. A non-significant decreasing effect of SB-mayo treatment on apoB concentrations (diff = −5.86 ± 18.26 mg/mL; *P* = 0.07) was noted compared to PO-mayo treatment, whereas apoA1 levels were not affected (*P* > 0.05).

**Table 4:** Metabolic parameters in response to mayonnaise diets

Parameters	Baseline	SB-mayo diet 28^th^ day	PO-mayo diet 28^th^ day	Difference between diets	*P-*_*time effect*_(SB-mayo)	*P-*_*time effect*_(PO-mayo)	*P*-_*diet differences*_(ES)
TC (mmol/L)	5.20 ± 0.56	4.80 ± 0.65	5.06 ± 0.61	−0.25 ± 0.40	<0.001	0.010	0.001 (0.63)
LDL-C (mmol/L)	3.37 ± 0.52	3.05 ± 0.57	3.22 ± 0.57	−0.17 ± 0.40	<0.001	0.020	0.016 (0.43)
HDL-C (mmol/L)	1.39 ± 0.27	1.21 ± 0.27	1.34 ± 0.30	−0.12 ± 0.15	<0.001	0.090	<0.001 (0.80)
TAG (mmol/L)	0.94 ± 0.36	1.13 ± 0.54	1.08 ± 0.45	0.05 ± 0.28	<0.001	0.010	0.285 (0.18)
VLDL-C (mmol/L)	0.51 ± 0.19	0.61 ± 0.29	0.58 ± 0.25	0.03 ± 0.15	<0.001	0.010	0.285 (0.20)
LDL-C:HDL-C	2.54 ± 0.72	2.67 ± 0.87	2.56 ± 0.80	0.12 ± 0.41	0.070	0.820	0.106 (0.29)
ApoA1 (mg/mL)	138 ± 20	126 ± 19	124 ± 19	2.00 ± 22.31	<0.001	<0.001	0.605 (0.09)
ApoB (mg/mL)	119 ± 23	111 ± 26	117 ± 25	−5.86 ± 18.26	0.040	0.700	0.070 (0.32)
Glucose (mmol/L)	4.76 ± 0.37	4.92 ± 0.32	4.95 ± 0.30	−0.04 ± 0.30	0.020	0.010	0.475 (0.13)
NEFA (mEq/L)	0.54 ± 0.19	0.49 ± 0.17	0.49 ± 0.15	0.00 ± 0.15	0.080	0.120	0.963 (0.00)
T-BARs (mg/mL)	1.19 ± 0.77	1.22 ± 0.79	1.15 ± 0.66	0.06 ± 0.53	0.840	0.820	0.483 (0.11)
hsCRP (mg/mL)	0.22 ± 0.28	0.16 ± 0.21	0.19 ± 0.21	−0.03 ± 0.28	0.240	0.600	0.492 (0.11)

Values are reported as mean ± SD of 34 subjects; significance is at *P* <0.05. All values were adjusted for change in body weight

*Abbreviations*: *TC* total cholesterol, *HDL-C* high density lipoprotein-cholesterol, *LDL-C* low density lipoprotein-cholesterol, *TAG* triaclyglycerol, *VLDL-C* very low density lipoprotein-cholesterol, *NEFA* non-esterified fatty acids, *T-BARs* thiobarbituric acid, *hsCRP* high sensitive C-reactive protein, *ES* effect size

### Lipoprotein particle measures

Table 5 provides distribution data for lipoprotein particle patterns analyzed for LDL and HDL particles for 31 subjects (3 subjects were excluded because plasma volume was insufficient). Dietary treatment did not significantly affect total HDL particle count (THP) or size distributions (*P* > 0.05).

**Table 5:** Lipoprotein particle count and lipoprotein particle size distribution as per dietary treatment

Particle size	Baseline	SB-mayo diet 28^th^ day	PO-mayo diet 28^th^ day	Difference between diets	*P*_*time effect*_(SB-mayo)	*P*_*time effect*_(PO-mayo)	*P*-_*diet differences*_(ES)
LDL particles (nmol/L)							
TL	1165 ± 318	1094 ± 327	1129 ± 310	−35.0 ± 169.4	0.050	0.190	0.259 (0.21)
LL	574 ± 137	500 ± 151	563 ± 143	−63.2 ± 122.2	0.010	0.540	0.007 (0.52)
IL	48.4 ± 31.7	27.5 ± 33.5	37.0 ± 26.0	−9.50 ± 24.61	<0.001	0.020	0.040 (0.39)
SL	543 ± 363	567 ± 377	530 ± 372	37.7 ± 227.8	0.010	0.540	0.365 (0.17)
		Mean LDL particle size (nm)					
	21.6 ± 0.70	21.4 ± 0.77	21.6 ± 0.71	−0.15 ± 0.48	0.190	0.750	0.095 (0.31)
HDL particles (μmol/L)							
TH	31.2 ± 3.71	28.7 ± 3.62	29.4 ± 3.28	−0.67 ± 2.56	<0.001	<0.001	0.154 (0.26)
HL	8.5 ± 2.98	7.8 ± 2.77	7.98 ± 2.66	−0.16 ± 1.45	0.090	0.080	0.558 (0.11)
HM	3.25 ± 2.09	2.46 ± 1.65	2.42 ± 2.42	0.04 ± 2.02	0.090	0.090	0.918 (0.02)
HS	19.5 ± 4.24	18.4 ± 4.06	19.0 ± 3.80	−0.56 ± 3.10	0.120	0.290	0.326 (0.18)
		Mean HDL particle size (nm)					
	9.2 ± 0.46	9.2 ± 0.44	9.2 ± 0.47	−0.05 ± 0.19	0.490	<0.001	0.155 (0.26)

Values are reported as mean ± SD of 31 subjects; significance is at *P* < 0.05. All values were adjusted for change in body weight

*Abbreviations*: *TL* total particle count for LDL, *IL* intermediate LDL, *LL* large LDL and *SL* small LDL particle concentrations, *TH* total particle count for HDL, *HL* large HDL, *HM* medium HDL and *HS* small HDL particle concentrations. *ES* effect size

However, dietary treatment effect was associated with LDL particle size distributions specific to intermediate LDL (IL) and large LDL (LL) without any change (*P* > 0.05) in total LDL particle count (TLP). Overall, significant reductions in LL (diff = −63.2 ± 122.2 nmol/L, *P* = 0.007) and IL (diff = −9.50 ± 24.61 nmol/L, *P* = 0.040) particles occurred as a result of SB-mayo treatment compared to PO-mayo treatment. Treatment effects on small LDL (SL) were not apparent. Alteration in LDL subclass composition however did not result in differences in mean LDL particle size due to treatments.

### Markers of oxidative stress

NEFA, T-BARs and hsCRP outcomes were not significantly different (*P* > 0.05) between dietary treatments.

## Discussion

The 20 g exchange of test mayonnaise effectively reflected either linoleic (18:2) or palmitic (16:0) acid availability with dietary cholesterol equal between both diets. As intentioned, plasma fatty acid composition reflected higher percentages of palmitic and oleic acids (18:1) when subjects consumed PO-mayo or a linoleic acid (18:2, LA) with the SB-mayo.

Significant reductions in TC and LDL-C concentrations were associated with the SB-mayo rotation compared to the control mayonnaise. It was possible that these reductions were attributed to the higher LA availability from the SB-mayo as it is well established that LA reduces plasma cholesterol concentrations [31, 32]. Concomitant with this approach, it is said that diets lower in SFA and greater in PUFA content result in better CVD outcomes [1, 32, 33]. Substitution with PUFA for SFA was shown to lower CVD risk in the Medical Research Council study [34], where a high PUFA-rich SB feeding (20.4 %-en) compared to SFA as control (PUFA at 4.4 % - en) resulted in reduced number of cardiac events (*rr* = 0.86, 95 % CI 0.61, 1.22). The Finnish Mental Hospital Study reported lower mortality when SB replaced regular SFA-containing dairy products in both men (*rr* = 0.55, 95 % CI 0.34, 0.88) [35] and women (*rr* = 0.64, 95 % CI 0.41, 1.00) [36]. These were intervention trials running for 6 years with PUFA provided at 12.9 % of total calories compared to its content in the control diet at 4.3 % of total calories in the present study. The St. Thomas’ Atherosclerosis Regression Study reported a displacement of SFA with PUFA resulted in reversing atherosclerosis with the greatest regression in those reporting consumption of the least amount of SFA [37]. The Atherosclerosis Risk in Communities Study indicated the highest quartile of circulating cholesteryl linoleate after the LA-enriched diet was associated with lower carotid artery intimal medial thickness [38].

Subjects on the PO-mayo nevertheless experienced a small reducing effect of TC and LDL-C from baseline which could be attributed to its higher oleic acid (18:1) content. Indeed percent oleic acid in plasma after completing the PO-mayo phase was greater (by ~3 %) compared to baseline or during the SB-mayonnaise rotation. MUFA coexists with SFA in many foods which would be true as indicated by the fatty acid composition of palm olein which contains on average 45 % of oleic acid and 39 % of palmitic acid compared to 22 and 11 % respectively for these fatty acids in SB [9]. Additionally, comparison of palm olein and olive oil in human feeding trials indicate that dietary palmitic and oleic acids exert similar effects on plasma lipoproteins [39, 40]. Reduction in dietary SFA intake is also associated with a fall in HDL-C concentrations depending on the substituting nutrient exchange [32]. LA’s additional role in lipoprotein metabolism is in mediating a reducing effect on HDL-C concentrations [31]. We observed this effect when subjects were consuming the LA-rich SB-mayo but not with the PO-mayo. However, LDL-C:HDL-C ratios were not affected. We also noted this HDL-C reduction did not affect total HDL lipoprotein particle number or size distributions.

The LA-enriched diet that included the SB-mayo did not result in an expected significant decrease in apoB concentrations. ApoB is a surrogate measure for atherosclerotic lipoprotein particles as more than 90 % of apoB is carried by LDL particles [41]. LDL lipoprotein subclass distribution is directly implicated in atherogenesis with reduced large buoyant LDL particles and increased small dense LDL particles favouring atherosclerotic plaque deposition [42, 43]. We investigated LDL lipoprotein particle distribution and found an overall 3 % decrease in total LDL particle translated into significant 19.6 % decrease in intermediate LDL particles and also an 11 % decrease in large LDL particles, without a significant change in small LDL particles when subjects were regularly consuming the SB-mayo. Previous interventions have reported standard [44] or high dose dietary cholesterol [45] feeding did not cause shifts in LDL particle size irrespective of cholesterolemic status. Thus it is probable that the shifts in LDL lipoprotein particle distributions observed in our study could be attributed to the enriched-LA diet which was exchanged for palmitic acid.

A major concern with higher LA consumption is the hypothesis it could promote an inflammatory environment central to chronic disease development [46]. One pathway is LA-induced arachidonic acid (20:4ω6) enrichment in tissue phospholipids would lead to excessive generation of pro-inflammatory eicosanoids [46, 47]. A systematic review examining Western-type diets did not find, after dietary manipulation, changes in plasma or serum arachidonic acid levels despite a 6-fold increase in dietary LA consumption or up to 90 % reduction in LA [47]. Our study too did not find this effect. Another systematic review of randomized controlled trials, assessing addition of LA to the diet of healthy non-infant populations, did not find any evidence that LA increased the concentration of biologic markers of chronic inflammation including CRP, fibrinogen, plasminogen activator inhibitor type 1, cytokines, soluble vascular adhesion molecules, or tumour necrosis factor-α [48]. We included only hsCRP as an inflammatory marker in our study but this marker was not significantly affected after consumption of the LA-rich diet enriched by the SB-mayo addition.

The capacity of mayonnaise to be conducive in the development of MetS through the inflammatory environment has been suggested [15]. We did not find any significant cause-effect changes in plasma glucose levels or oxidative stress parameters of exogenous origin such as NEFA and T-BARs in response to the dietary manipulation supplementing 20 g of mayonnaise consumption daily of either SB- or PO-based formulations. Our findings are not in agreement with the observations of Akter et al. [13], who reported mayonnaise consumption, was positively associated with high blood glucose in an adult free living Japanese population with MetS. This difference may in part be explained by the normoglycemic status of our subjects when recruited into the trial.

Donovan and Shamir [49] view the human diet as a complex food matrix rather than specific nutrients, and highlight a need to resolve how to term a food’s quality or necessity judged as part of an individual’s whole diet. Citing yogurt as an example, they defined its potential as a functional food to manage lactose intolerance or bone health, despite concerns about its high SFA content, which is implicated in the promotion of heart disease. The potential for mayonnaise to be a vehicle for nutraceutical delivery has been explored with diacylglycerol in an anti-obesity function or with plant sterol esters for decreasing LDL-C [50, 51]. Saito et al. [51] who examined the dose-dependent effect of a mayonnaise-type product combining diacylglycerol with plant sterol esters in subjects with mild hypercholesterolemia found daily ingestion of 15 g of diacylglycerol plus mayonnaise containing at least 0.4 g/d of plant sterol esters for 4 weeks significantly decreased serum cholesterol levels. This load of mayonnaise was similar to the planned intervention in our study.

Results of our study suggest that the active component of SB-mayo is linoleic acid which was observed to lower plasma LDL-C levels. Currently, mayonnaise is a condiment that is commercially available as a general food product and excessive consumption could lead to increased lipid intake which would be counterproductive in terms of excess calories. As scientific proof is necessary to establish the utilization of mayonnaise as a nutraceutical, further research is necessary to determine the appropriate serving size of a mayonnaise product that could lower serum LDL-C levels.

The public health approach for heart health stresses a ~50 % reduction in SFAs to effect a reduction in LDL-C as a means to decrease risk of CVD [1]. Quantitatively, lowering SFAs by 50 % requires substitution with food sources of ω-6 and ω–3 PUFAs and MUFAs [1, 4, 33]. Weighing the emerging nutrition science on fatty acids and CVD, Kris-Etherton and Fleming [33] acknowledge the best evidence for cardioprotective benefits from substitution of SFAs remains with ω–6 PUFAs, as evidence for MUFAs is mixed whilst only relatively low amounts of ω-3 PUFAs can be incorporated in the diet, even with very high dose. Through this study we have provided evidence that an LA-rich mayonnaise is a convenient vehicle for nutraceutical delivery of the ω-6 PUFA, linoleic acid, therefore expecting a functional use in reduction of LDL-C as a means to decrease risk of CVD. The extrapolation of this study findings may be limited to the general free living population because of the small number of subjects (*n* = 34) who were relatively young and required to consume research diets provided in an institutional setting.

## Conclusions

We demonstrated a functional role for an SB-mayo which was rich in linoleic acid. Uptake of linoleic acid was enhanced, accompanied by a demonstration of the biological activities of increased ω-6 PUFA linoleate. This was also achieved without detrimental shifts in the overall plasma oxidative status of the subjects. These outcomes suggest that the inclusion of about 20 g linoleic acid -rich mayonnaise could be managed as part of a daily healthy diet given the current modern lifestyles that often dictate consumption of prepared foods on the go.

## Abbreviations

Apo, apolipoprotein; FAC, fatty acid composition; HDL-C, high density lipoprotein cholesterol; hsCRP, high sensitivity C-reactive protein; LA, linoleic acid; LDL-C, low density lipoprotein cholesterol; MetS, metabolic syndrome; MUFA, mono unsaturated fatty acid; NEFA, non-esterified fatty acids; NMR, nuclear magnetic resonance; PO, palm olein; PUFA, poly unsaturated fatty acid; SB, soybean oil; SFA, saturated fatty acid; T-BARs, thiobarbituric acid reactive substances; TC, total cholesterol; TG, triglycerides; VLDL, very low density lipoprotein cholesterol. Lipoprotein particles: TL, total particle count for LDL; IL, intermediate LDL; LL, large LDL and SL, small LDL particle sizes; TH, total particle count for HDL; HL, large HDL; HM, medium HDL and HS, small HDL particle size
